# Supplementary figures and images for: RNA editing at a limited number of sites is sufficient to prevent MDA5 activation in the mouse brain
Source: PLoS Genet. 2021 May 13;17(5):e1009516. doi: 10.1371/journal.pgen.1009516 (PMC8118328; doi:10.1371/journal.pgen.1009516)

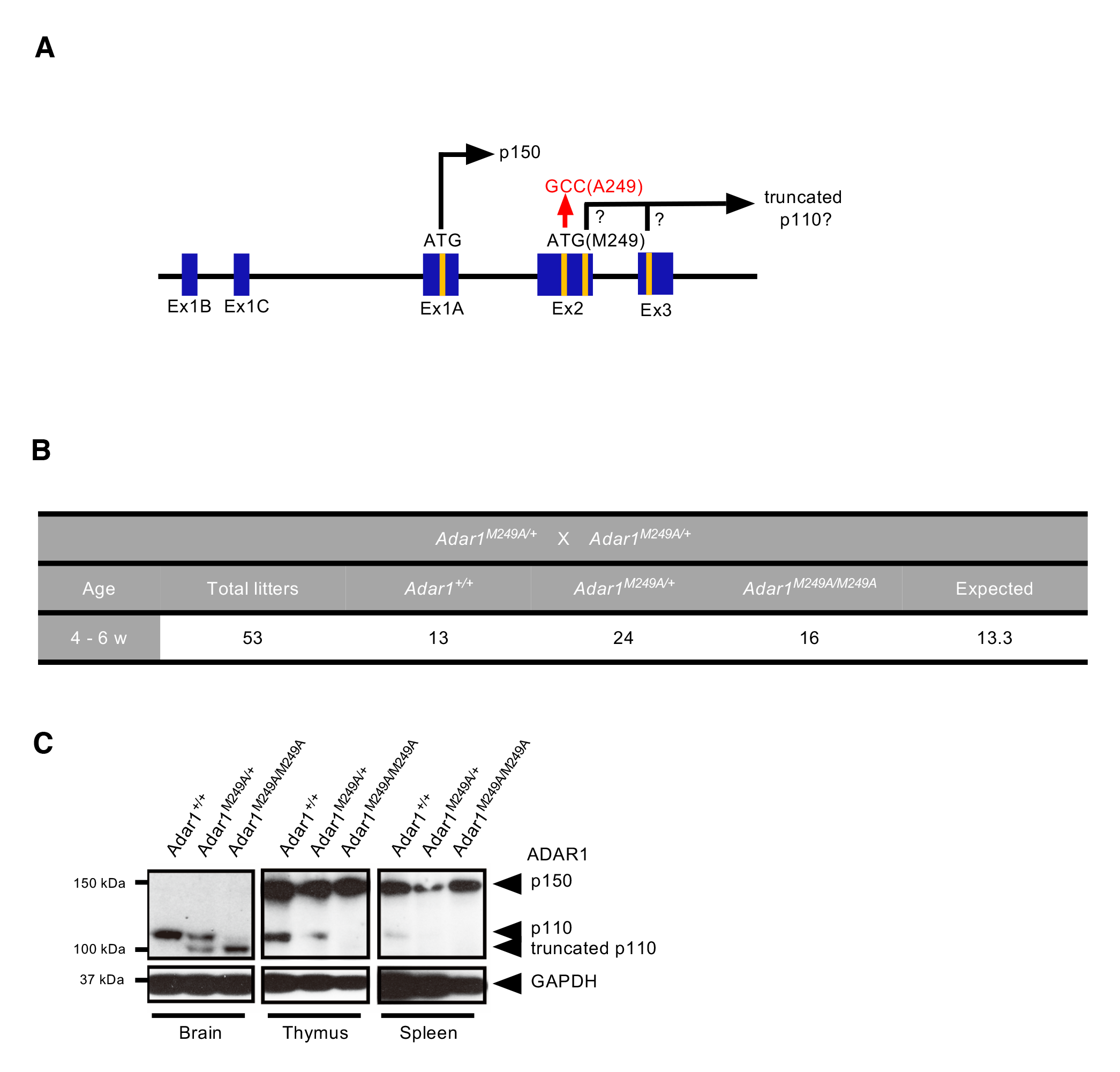

Supplement: S1 Fig — (A) Schematic diagram of mouse Adar1 gene. A point mutation was inserted at the position of amino acid 249 from ATG to GCC in exon 2 (Ex2), which converted the initiation methionine (M249) to alanine (A). This conversion was expected to specifically inhibit translation of the ADAR1 p110 isoform but instead induced the expression of a truncated p110 isoform translated from the downstream methionine most likely located in either Ex2 or Ex3. (B) The number of the surviving littermates of wild-type (Adar1+/+), Adar1M249A/+, and Adar1M249A/M249A mice between 4 to 6 weeks of age. (C) Immunoblot analysis of ADAR1 p110 and p150 protein expression in brains, thymi, and spleens of Adar1+/+, Adar1M249A/+, and Adar1M249A/M249A mice. The expression of GAPDH protein is shown as a reference. (TIF) [file pgen.1009516.s001.tif]

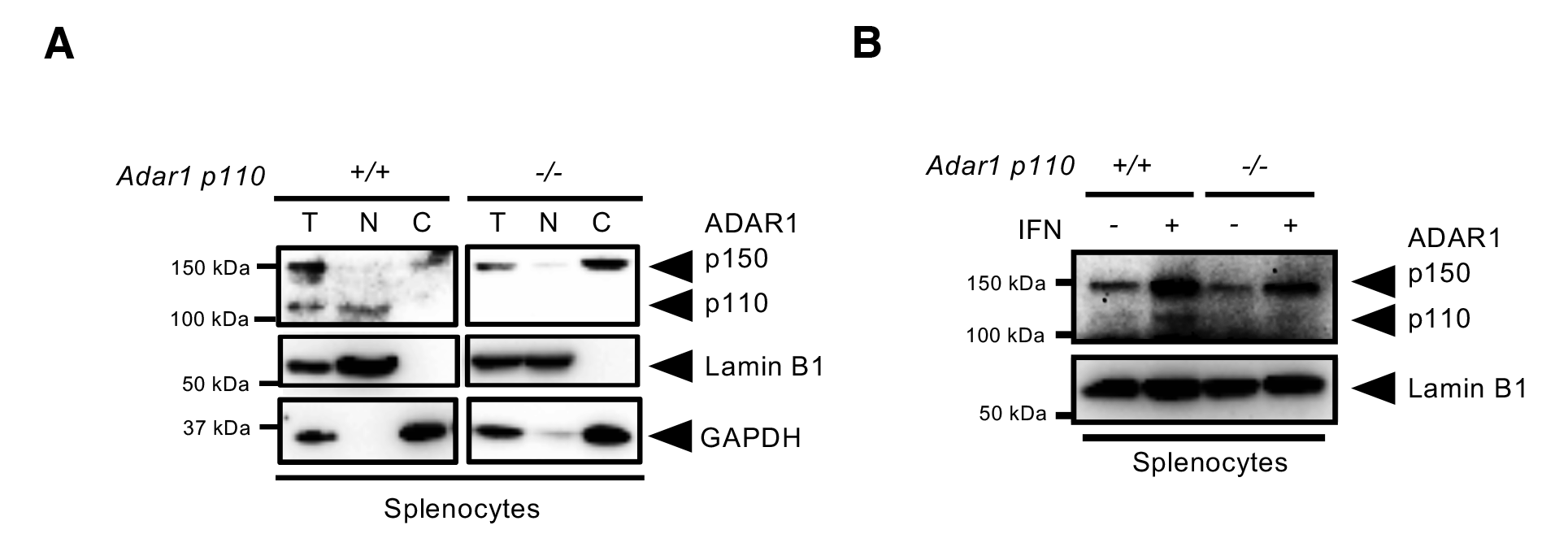

Supplement: S2 Fig — (A) Immunoblot analysis of ADAR1 p110 and ADAR1 p150 protein expression in total lysates (T), nuclear fraction (N), and cytoplasmic fraction (C) of splenocytes isolated from wild-type (Adar1 p110+/+) and Adar1 p110–specific knockout (KO; Adar1 p110-/-) mice. The expression of Lamin B1 and GAPDH proteins is shown as nuclear and cytoplasmic markers, respectively. (B) Immunoblot analysis of ADAR1 p110 and ADAR1 p150 protein expression in splenocytes isolated from Adar1 p110+/+ and Adar1 p110-/- mice. Splenocytes were cultured in the absence (-) or the presence (+) of interferon (IFN)-β1 stimulation for 20 h. The expression of Lamin B1 protein is shown as reference. (TIF) [file pgen.1009516.s002.tif]

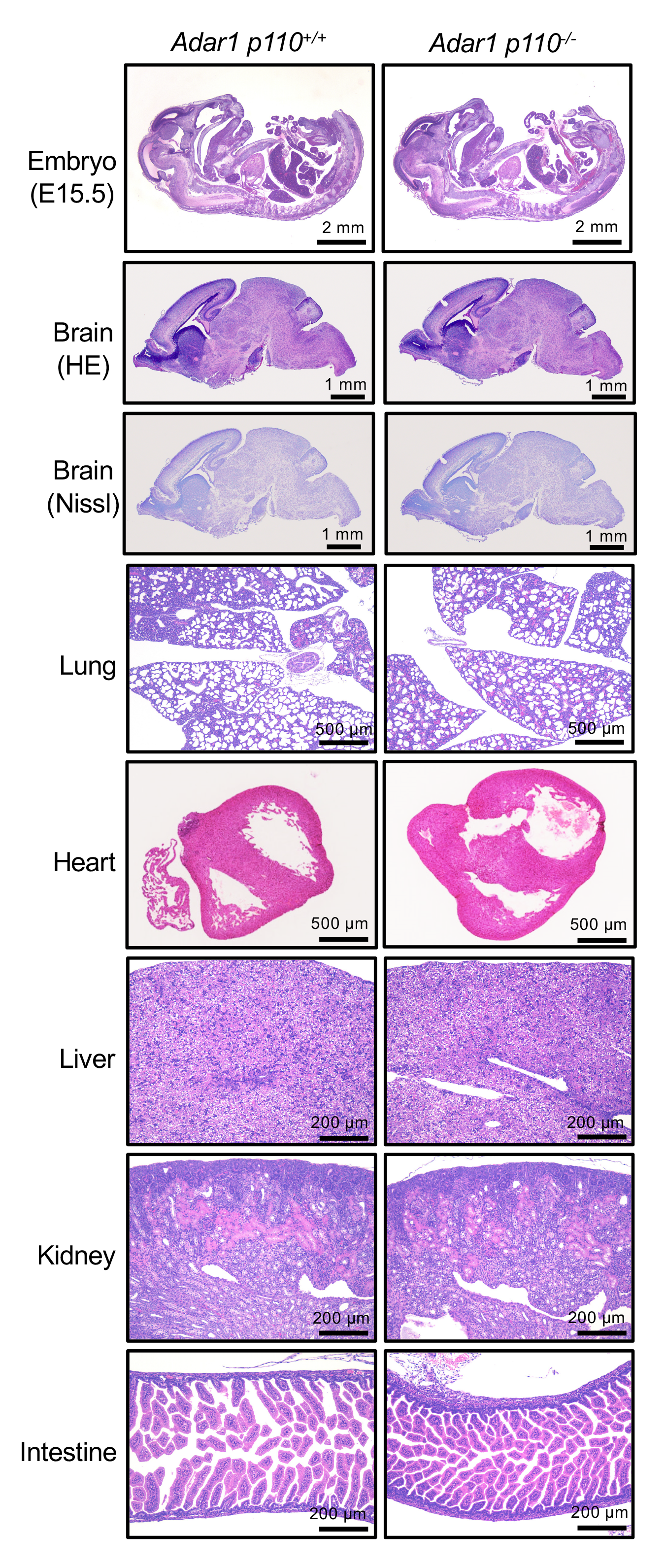

Supplement: S3 Fig — Representative images of hematoxylin and eosin (HE) staining of the whole embryo at E15.5 and the brain, lung, heart, liver, kidney and intestine at post-natal day 0 (P0), and those of Nissl staining of the brain at P0 from wild-type (Adar1 p110+/+) and Adar1 p110–specific knockout (Adar1 p110-/-) mice. The scale bar is indicated in each panel. (TIF) [file pgen.1009516.s003.tif]

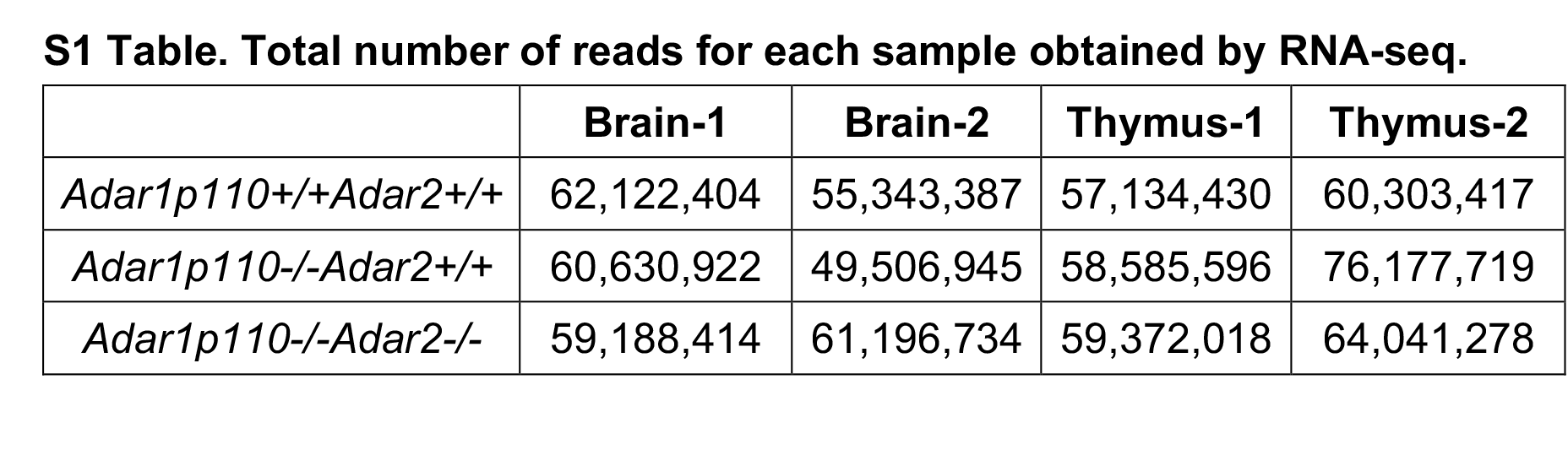

Supplement: S1 Table — (TIF) [file pgen.1009516.s004.tif]

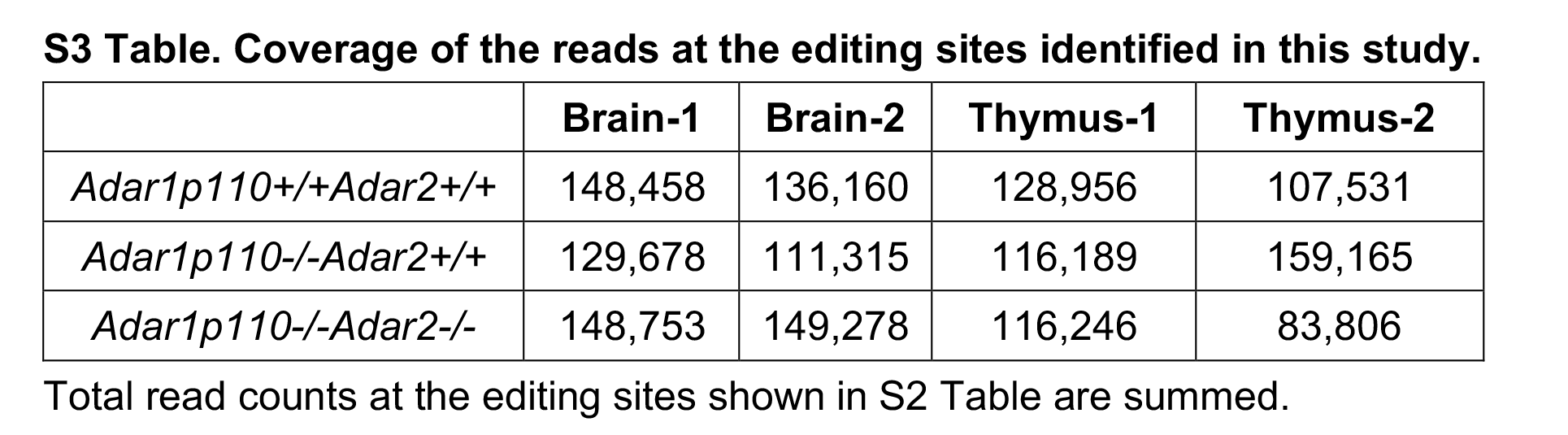

Supplement: S3 Table — (TIF) [file pgen.1009516.s006.tif]
