# Supplementary material for: RNA editing at a limited number of sites is sufficient to prevent MDA5 activation in the mouse brain
Source: PLoS Genet. 2021 May 13;17(5):e1009516. doi: 10.1371/journal.pgen.1009516 (PMC8118328; doi:10.1371/journal.pgen.1009516)
Supplement: Attachment — Submitted filename: Kim_Responses to the review comments.pdf [file pgen.1009516.s011.pdf]

We thank all reviewers for their careful evaluation of our manuscript. We have tried to incorporate all comments and suggestions into the manuscript, which have enabled us to significantly improve its scientific content.

Answers for Reviewer #1:

In this study by Kim et al., the authors generate different ADAR1 p110 knockout mice and assess A->G editing in the transcripts. As there is very little expression of p150 isoform in the brain, the authors propose that p110 may be involved in the editing of endogenous dsRNA and preventing MDA5 activation. First, the author generate a p110 specific KO mice by mutating the start codon M294. Unfortunately, this resulted in the expression of a truncated form of p110, likely due to translation initiation from an internal AUG. Next, the authors generate another version of p110 KO mice by deleting the constitutive ADAR1 promoter along with Exon1B and 1C, and successfully derive mice lacking p110; however, the majority of p110-/- mice die within 2 days after birth. Unlike ADAR1-/- or p150-/-, p110-/- mice do not exhibit increased expression of interferon related genes. This early lethality can be rescued by expressing editing mutant of ADAR1E861A but not by concurrent deletion of MDA5, suggesting that early death is unrelated to RNA editing functions of p110. Using this novel p110-/- mice, the authors investigated the alterations A->G mutations in the transcriptome and observed only partial reduction in A->G mutations. Through concurrent deletion of p110-/- and ADAR2-/-, the authors demonstrate that both p110 and ADAR2 are contributing to A->G mutations. The authors also identify a few remaining mutations that may be attributed to p150 but not conclusively. This is a novel and well executed study. The manuscript is also well written. I have some minor comments.

We thank the reviewer for these comments.

1. For p110-/- mice, the authors indicate that the contributions of p110 expressed from a cryptic promoter or from p150 mRNA through the internal M294 is minimal. The authors should treat p110-/- cells with IFN treatment and assess p110 expression, as previously suggested for human cells (Cell. 2018;172(4):811-24.e14.; https://doi.org/10.1371/journal.ppat.1003963; https://doi.org/10.1371/journal.ppat.1008842).

Thank you for this insightful comment. In response to this suggestion, we treated splenocytes isolated from adult *Adar1 p110*$^{-/-}$ mice with IFN-β1. This analysis has demonstrated that ADAR1 p110 expression was not detectable in *Adar1 p110*$^{-/-}$ splenocytes even when ADAR1 p150 protein expression was upregulated (**S2B Fig**), suggesting that ADAR1 p110 is not efficiently produced from a cryptic promoter and Adar1 p150 mRNA in mice, which might be different from the phenomena reported in human cell lines. We described this result in the Result section (**Page 12, lines 190-193**) and discussed the difference by citing three papers indicated by the reviewer (**Page 26, lines 396-400**) in addition to the methods used for this study (**Page 33, line 501 – Page 34, lines 508**).

2. The authors should also assess editing in ADAR1E861A/p110-/- mice to rule out any residual editing by E861A mutant.

Thank you for this suggestion. We previously reported that no editing events were detected in *Adar1*$^{E861A/E861A}$ *Adar2* KO mice (Cruz et al, RNA, 2000), indicating that E861A mutant completely loses its editing activity (**Page 6, lines 85-87**). Therefore, it is anticipated that ADAR1 p150 that is expressed from one allele is the sole active ADAR1 isoform present in *Adar1*$^{E861A/p110del}$ mice. In contrast, given that ADAR2 is present in these mutant mice, we assessed the editing of only the sites shown in Fig 9, which are likely ADAR1 p150–specific sites. As shown in the new **Fig 9**, the editing of these sites was preserved in the brain of *Adar1*$^{E861A/p110del}$ mice, which further suggests that a subtle amount of ADAR1 p150 is sufficient to sustain the editing ratio of certain sites. We have added a description of this result to the main text (**Page 22, lines 345-347**).

3. It will be informative to the readers if the authors could include A->G editing in the repeat sequences (SINE). This could provide information to readers about the levels of p150 editing.

Thank you for this comment. We had already categorized all the editing sites into the repeat sequences (shown as “repetitive”) and non-repeat sequences (“non-repetitive”) in **S2 Table**.

4. LN 39- p is missing in p150

Thank you for reminding us of this mistake. We have fixed the indicated point (**Page 3, line 39**).

Answers for Reviewer #2:

The ADAR family of RNA editing enzymes are important for modulating the structure and coding properties of the mammalian transcriptome. Here the authors describe the generation and characterization of mice genetically modified to express only the p150 isoform of ADAR1 and not the p110 isoform of ADAR1 or ADAR2. Since these three proteins are the only editing-functional mammalian ADARs, these mice provide an important tool for the study of phenotypes associated with p150 and p150-specific editing sites. Other labs have shown that ADAR1 p150 activity is essential to block recognition by duplex RNA sensors (e.g. MDA5) of the duplex RNA structure present in the transcriptome. However, a critical question that has been dogging the editing community is "What is the identity of the critical RNAs modified by ADAR1 that would otherwise by recognized by dsRNA receptors and trigger and immune response?" This study provides key data that start to address this question. By removing the other functional ADARs from the mouse genome, these authors can identify the sites edited by ADAR1 p150. The results reported in Figure 9 described highly efficient ADAR1 p150 editing sites, particularly in the 3' UTR of the Mad2ll transcript, are very important. The conclusions of this paper are supported well by the results presented. I have only the following minor comments.

We thank the reviewer for these comments.

1) The introduction is quite wordy with the last paragraph essentially providing a synopsis of the entire study. The authors may wish to move some of this text to the discussion.

Thank you for this comment. We have shortened the last paragraph in the Introduction from 27 sentences to 10 sentences (**Page 9, lines 138-141**).

2) The discussion of the truncated ADAR1 p110 found in the M249A/M249A mice

is distracting and does not add to the overall impact of the paper. The authors may consider removing this entirely from the manuscript.
Thank you for pointing this out. In response, we have deleted the corresponding sentence (Page 24, lines 372-374 in the original manuscript).

3) An obvious question for these authors to address is whether edited Mad2ll 3'-UTR interacts with dsRNA sensor proteins like MDA5 differently than the unedited RNA. While I don't believe these results are essential for publication, they could substantially raise the impact of the current work.
Thank you for this insightful comment. We agree that the experiment proposed by the reviewer is crucial as a next step to know how differently edited and unedited transcripts bind to and activate MDA5. However, as described in the Discussion part (**Page 27, line 419 – Page 28, line 436**), although 3'UTR of Mad2l1 is a prime candidate site, we currently have to continue identification of highly edited sites and determine which editing sites are critical for preventing MDA5 activation. Therefore, we would like to perform the suggested experiment as a separate study in the future. Thank you again for this comment.

Answers for Reviewer #3:
Summary
The paper directly by Kim et al. addresses the different roles for ADAR p150 and p110 isoforms, not easy given the given the complexity of dsRNA dependent pathways. In a technical tour de force, Kim et al. succeeded in creating a mouse that expressed only p150, but no detectable p110. This approach enabled the authors to address longstanding questions in the field concerning the roles played by p150 and p110 isoforms, the cytoplasmic substrates edited by p150 and why there is rescue by Mavs5 null alleles of the perinatal mortality caused by the p150 isoform knockout. A milestone effort! The only addition to the paper necessary is a direct demonstration that the p150 isoform in the p110 null mouse is mostly cytoplasmic.
Findings
1.Adar1 p110–specific knockout mice do not upregulate expression of ISGs
2.Less than 2% of editing sites in brain are ADAR1 p150–mediated in the Adar1

p110/Adar2 double knockout mice.

3.The observed edits are mostly in the 3' UTR, although editing of an Azin1 exon is reported. The findings are consistent with cytoplasmic editing by p150 in their p110 null mouse.

4.Editing level of ADAR1 150–specific sites is similar in Adar1 p110/Adar2 double knockout mice and wildtype mice.

5.The high mortality rate during the early post-natal stages observed in Adar1 p110-specific KO mice is rescued by the expression of catalytically inactive Adar1 in Adar1E861A/p110del mice, but not by Ifhi1 deletion.

6.There is no elevated expression of ISGs was observed in Adar1 p110-/- Adar2-/- mice at P0.

7.The Adar1 constitutive promoter is dispensable for regulating the expression of Adar1 p150 and loss of p110 does not upregulate expression of p150 and also indicates that there is no interferon induction in this model.

We thank the reviewer for these comments. In particular, the comment “A milestone effort!” is very encouraging for us.

Major Comment

The p150 is known to undergo nuclear-cytoplasmic shuffling. While the editing dat presented by the authors is consistent with only a cytoplasmic localization of p150 in the p110 null mouse, it is necessary to show this. The reasons are twofold. Firstly, the nuclear and cytoplasmic localization of the p150 Zα mutant mouse described on BioRxiv by the Maelfait group is wildtype. Second, the non-editing role of p110 may be to promote nuclear uptake of p150 by sponging up the dsRNA that inhibits the bipartite the ADAR nuclear localization signal. In the absence of p110, dsRNA accumulation in the cytoplasm could prevent p150 nuclear uptake (see PMID: 24753571).

A low nuclear uptake may also account for the lack of editing in the brain. Important events may be missed if p150 disease relevant edits only occur in the nucleus. The possibility that nuclear editing by p150 is certainly consistent with the other paper the Kawahara group has on BioRxiv in which the editing ratio of ~30% of sites in AdarW197A/W197A mice was more than 10% higher than that in Adar1+/+ mice, while the editing ratio of ~5% of sites in Adar1W197A/W197A mice

was more than 10% lower than that in Adar1+/+ mice.

I hope the experiments to confirm the cytoplasmic localization of p150 in this model won't delay publication as the question can be quickly settled by immunofluorescence or cell fractionation approaches.

Thank you for this insightful comment. In response, we performed subcellular fractionation for splenocytes isolated from *Adar1 p110*$^{-/-}$ mice. We chose splenocytes because ADAR1 p150 is abundantly expressed in addition to the distinct expression of ADAR1 p110, whereas ADAR1 p150 is expressed at very low levels in the brain. As expected, this analysis revealed that ADAR1 p150 was predominantly detected in the cytoplasmic fraction of ADAR1 p110-deficient splenocytes (**S2A Fig**), indicating that loss of ADAR1 p110 does not largely affect the intracellular localization of ADAR1 p150. We described this result in the Result section (**Page 12, lines 189-190; Page 13, lines 197-199**) in addition to the methods used for this study (**Page 34, line 518 – Page 35, lines 530; Page 36, line 541**).

Minor Comments

1.Line 64 "domain α (Zα domain), is largely expressed in the cytoplasm [15-19]." I think it is more accurate to say that it undergoes nucleocytoplasmic shuffling, with accumulation in the cytoplasm observed under conditions of high expression, such as occurs during viral infections, using the references from line 74 "which might shuttle between the nucleus and cytoplasm [15, 17, 18]." Reference 16 shows that p150 can be highly expressed in the nucleus after interferon induction.

Thank you for this comment. We have changed the corresponding sentences in accordance with your suggestion (**Page 5, line 80 – Page 6, line 19**).

2.Line 126 "This analysis demonstrated that more than 98% of all the editing sites found in wild-type mice were absent in the brain" while in line 131, it is stated that "the number of editing sites was less than 10% of that found in wild-type mice" give different percentages. Does the line 131 include editing observed in all tissue?

Thank you for this comment. In contrast to the number "98%", which was calculated based on the total editing sites, the number "10%" was the proportion based on the editing sites only in 3'UTR. In response to the comment 1 from Reviewer #2, however,

we have removed these sentences to shorten the Introduction part.

3.Line 129 "indicating that ADAR1 p150 does not substantially contribute to RNA editing in the nucleus". This may only be due to a lack of nuclear import in the p110 null mouse (see comments above).

Thank you for this comment. We performed an additional experiment (**S2A Fig**) in response to this comment; please refer to our response to your Major Comment. We should note that although the sentence in Line 129 of the original manuscript has been removed from the Introduction part in response to the comment 1 from Reviewer #2, the similar sentence can be found in **Page 26, lines 402-403** in the Discussion part.

4.I think the wording in the sentence starting on line 212 may need rewriting. "The resulting Adar1E861A/p110del mice demonstrated no obvious abnormal phenotypes that differed from those in Adar1 p110-/- mice or the embryonic lethality found in Adar1E861A/E861A mice (Fig 2I)." instead of "that differed" do the authors mean ", an outcome different from"….

Thank you for this comment. We have modified the corresponding sentence in accordance with your suggestion (**Page 14, line 222**).

5.I think the wording on line 923 "the number of the sites commonly identified in two mice" may need rewriting. Do the authors mean "the number of sites in common between the two mice studied"?

Thank you for this comment. In response, we have revised the sentence to "the number of sites in common between the two mice studied" (**Page 66, line 1010**).

6.Legend to Fig. 2I does not describe the data shown: "901 Scale bar, 1 cm. (I) Survival curves of Adar1 p110+/+ Ifih1-/- (n = 11), Adar1 p110+/- Ifih1-/- (n = 20), and Adar1 p110-/- Ifih1-/- mice (n = 7)."

Thank you for reminding us of this mistake. We have fixed the indicated point (**Page 65, lines 988-989**).

7.A better reference for the statement "In addition, another mutation is found in the p150 isoform–specific Zα domain, which indicates that the reduced RNA

editing activity of ADAR1 p150 is probably a cause of AGS pathogenesis" is PMID: 31320745 where there is direct evidence that p150 mutations do cause AGS.

Thank you for this comment. In response, we have cited the suggested paper (Herbert et al., Eur J Hum Genet, 2020) in this sentence (**Page 29, lines 448-450**).
