# Supplementary material for: RNA editing at a limited number of sites is sufficient to prevent MDA5 activation in the mouse brain
Source: PLoS Genet. 2021 May 13;17(5):e1009516. doi: 10.1371/journal.pgen.1009516 (PMC8118328; doi:10.1371/journal.pgen.1009516)
Supplement: Attachment — Submitted filename: PGENETICS-D-20-01959 p110 null phenotype Kim et al 2021 V1.docx [file pgen.1009516.s009.docx]

**RNA editing at a limited number of sites is sufficient to prevent MDA5 activation in the mouse brain**

**Authors:** Jung In Kim, Taisuke Nakahama, Ryuichiro Yamasaki, Pedro Henrique

6 Costa Cruz1, Tuangtong Vongpipatana, Maal Inoue, Nao Kanou, Yanfang Xing, Hiroyuki Todo, Toshiharu Shibuya, Yuki Kato, Yukio Kawahara

**PGENETICS-D-20-01959**

**Reviewer:** Alan Herbert

**Summary**

The paper directly by Kim et al. addresses the different roles for ADAR p150 and p110 isoforms, not easy given the given the complexity of dsRNA dependent pathways. In a technical tour de force, Kim et al. succeeded in creating a mouse that expressed only p150, but no detectable p110. This approach enabled the authors to address longstanding questions in the field concerning the roles played by p150 and p110 isoforms, the cytoplasmic substrates edited by p150 and why there is rescue by *Mavs5* null alleles of the perinatal mortality caused by the p150 isoform knockout. A milestone effort! The only addition to the paper necessary is a direct demonstration that the p150 isoform in the p110 null mouse is mostly cytoplasmic.

**Findings**

1. Adar1 p110–specific knockout mice do not upregulate expression of ISGs
2. Less than 2% of editing sites in brain are ADAR1 p150–mediated in the Adar1 p110/Adar2 double knockout mice.
3. The observed edits are mostly in the 3' UTR, although editing of an Azin1 exon is reported. The findings are consistent with cytoplasmic editing by p150 in their p110 null mouse.
4. Editing level of ADAR1 150–specific sites is similar in Adar1 p110/Adar2 double knockout mice and wildtype mice.
5. The high mortality rate during the early post-natal stages observed in Adar1 p110-specific KO mice is rescued by the expression of catalytically inactive Adar1 in Adar1^E861A^/^p110del^ mice, but not by *Ifhi1* deletion.
6. There is no elevated expression of ISGs was observed in Adar1 p110-/- Adar2-/- mice at P0.
7. The Adar1 constitutive promoter is dispensable for regulating the expression of Adar1 p150 and loss of p110 does not upregulate expression of p150 and also indicates that there is no interferon induction in this model.

**Major Comment**

The p150 is known to undergo nuclear-cytoplasmic shuffling. While the editing dat presented by the authors is consistent with only a cytoplasmic localization of p150 in the p110 null mouse, it is necessary to show this. The reasons are twofold. Firstly, the nuclear and cytoplasmic localization of the p150 Zα mutant mouse described on BioRxiv by the Maelfait group is wildtype. Second, the non-editing role of p110 may be to promote nuclear uptake of p150 by sponging up the dsRNA that inhibits the bipartite the ADAR nuclear localization signal. In the absence of p110, dsRNA accumulation in the cytoplasm could prevent p150 nuclear uptake (see PMID: 24753571).

A low nuclear uptake may also account for the lack of editing in the brain. Important events may be missed if p150 disease relevant edits only occur in the nucleus. The possibility that nuclear editing by p150 is certainly consistent with the other paper the Kawahara group has on BioRxiv in which the editing ratio of ~30% of sites in *Adar*^W197A/W197A^ mice was more than 10% higher than that in Adar1^+/+^ mice, while the editing ratio of ~5% of sites in *Adar1*^W197A/W197A^ mice was more than 10% lower than that in *Adar1*^+/+^ mice.

I hope the experiments to confirm the cytoplasmic localization of p150 in this model won’t delay publication as the question can be quickly settled by immunofluorescence or cell fractionation approaches.

**Minor Comments**

1. Line 64 “domain α (Zα domain), is largely expressed in the cytoplasm [15-19].”

I think it is more accurate to say that it undergoes nucleocytoplasmic shuffling, with accumulation in the cytoplasm observed under conditions of high expression, such as occurs during viral infections, using the references from line 74 “which might shuttle between the nucleus and cytoplasm [15, 17, 18],” Reference 16 shows that p150 can be highly expressed in the nucleus after interferon induction.

2. Line 126 “This analysis demonstrated that more than 98% of all the editing sites found in wild-type mice were absent in the brain” while in line 131 it is stated that “the number of editing sites was less than 10% of that found in wild-type mice” give different percentages. Does the line 131 include editing observed in all tissue?
3. Line 129 “indicating that ADAR1 p150 does not substantially contribute to RNA editing in the nucleus”. This may only be due to a lack of nuclear import in the p110 null mouse (see comments above).
4. I think the wording in the sentence starting on line 212 may need rewriting . “The resulting Adar1E861A/p110del mice demonstrated no obvious abnormal phenotypes that differed from those in Adar1 p110-/- mice or the embryonic lethality found in Adar1E861A/E861A mice (Fig 2I).” instead of “that differed” do the authors mean “, an outcome different from”….
5. I think the wording on line 923 “the number of the sites commonly identified in two mice” may need rewriting. Do the authors mean “the number of sites in common between the two mice studied”?
6. Legend to Fig. 2I does not describe the data shown : “901 Scale bar, 1 cm. (I) Survival curves of Adar1 p110+/+ Ifih1-/- (n = 11), Adar1 p110+/- Ifih1-/- (n = 20), and Adar1 p110-/- Ifih1-/- mice (n = 7).”
7. A better reference for the statement “In addition, another mutation is found in the p150 isoform–specific Zα domain, which indicates that the reduced RNA editing activity of ADAR1 p150 is probably a cause of AGS pathogenesis” is PMID: 31320745 where there is direct evidence that p150 mutations do cause AGS.
