# Supplementary material for: RNA editing at a limited number of sites is sufficient to prevent MDA5 activation in the mouse brain
Source: PLoS Genet. 2021 May 13;17(5):e1009516. doi: 10.1371/journal.pgen.1009516 (PMC8118328; doi:10.1371/journal.pgen.1009516)
Supplement: Attachment — Submitted filename: Perspective.docx [file pgen.1009516.s010.docx]

**To Z or not to Z: Self Recognition and Z-RNA formation**

Alan Herbert

**The background**:

The role of left-handed Z-DNA structure in biology has remained a mystery since its discovery. The initial description of this structure came from the first ever DNA crystal and was a surprise [1]. Its existence though had been anticipated by the observations of Pohl and Jovin [2], who noted an inversion of the optical properties of a ploy(dG-dC) DNA when it was placed in 5M salt, consistent with formation of a left-handed helix. After its atomic description, work in the Wang lab showed that Z-DNA should form from B-DNA without strand cleavage under conditions of topological stress, such as is generated during DNA transcription into RNA [3]. The next step was the identification of the Z-DNA specific, Zα binding domain in the double-stranded RNA (dsRNA) editing enzyme ADAR1 (encoded by *Adar* in mice) [4]. This enzyme deaminates adenosine to form inosine, which is subsequently processed by the cellular machinery as guanosine [5]. The focus at that time was on the recoding of mRNAs to produce new protein isoforms, the first example of which was the replacement of glutamine in the ion channel of the Gria2 receptor with arginine to change its conductance properties [6]. Subsequent work attributed the recoding to a related enzyme ADAR2 (encoded by *Adarb1*), that lacked a Z-binding domain. *Adarb1* knockouts had no phenotype in mice when an edited Gria2 allele was hardwired into the genome [7]. The story with ADAR1 was more complicated from the start. The gene encoded two isoforms: p150 with the Z-DNA binding domain that is induced by interferon and the constitutively expressed p110 (Fig 1A) [8]. The cellular location of the two isoforms varied. The p150 variant undergoes nucleocytoplasmic shuffling, whereas p110 is mostly nuclear, with cytoplasmic accumulation occurring only when bipartite the nuclear localization signal spanning the 3^rd^ dsRNA binding domain is blocked by dsRNA [9] or phosphorylated by stress kinases [10].

As methods to analyze RNA transcriptomes evolved, it became apparent that transposable elements (TEs) were the major ADAR substrates, with few recoding events. The editing substrates most often arose from pairs of TE repeats orientated in opposite directions, enabling then to form dsRNA by base-pairing with each other [11, 12]. The TE were most often SINES (short interspersed nuclear repeats) from the Alu family (named for the restriction enzyme that cuts twice within them) [13]. The question was what was the function of all this editing.

*Adar* knockout mice, or a loss of editing function variant, are embryonic lethal by day 14 [14]. The embryos have a type I interferon signature due to activation of the dsRNA sensor MDA5 (melanoma differentiation-associated gene 5, encoded by *Ifih1*) helicase that signals through the MAVS (mitochondrial antiviral signaling protein encoded by *Mavs*) pathway to induce interferon responses. Knockout of both *Adar* and *Ifih1* or *Mavs* provided partial rescue, with death a few days after birth [15, 16]. Quite surprising was the complete rescue of an editing null *Adar* variant by the *Ifih1* knockout [15]. The only reported phenotype was a delay in growth that was resolved within 12 weeks after birth. The results suggested that the function of ADAR1 was to inactivate endogenous dsRNAs that trigger the interferon pathway though MDA5, and that recoding by ADAR1 played no role in development. Knockout of the p150 by deletion of *Adar* exon 1a, leaving expression of p110 intact, also resulted in an interferon signature and neonatal death [17]. Unlike Adar null mice, most p150 deficient mice were completely rescued by *Mavs* deletion [16, 18], providing the initial evidence that p150 and p110 isoforms have different roles.

In humans, evidence that ADAR1 regulates interferon responses was provided by genetic studies in families with the Aicardi Goutières Syndrome Type I Interferonopathy [19]. Here deaminase domain loss of function variants produce an autosomal recessive disease. A role for the ADAR1 Zα in this disease was confirmed by an extended analysis of the Zα P193A variant reported in the initial report [20]. The identification of compound heterozygotes with one null allele allowed mapping of the P193A variant and another N173S variant directly to disease, exploiting the fact that the transcriptome was haploid in affected individuals [20]. Previous *in vitro* mutational scanning had identified both residues as essential to Z-DNA binding [21], findings supported by the Zα-Z-DNA crystal and NMR structures [22, 23] and then by the structure of Zα bound to left-handed Z-RNA [24]. Separate studies also showed many cancers depend on p150 to silence immune responses [25].In three recent studies with mouse models, loss of function mutations of other Zα residues involved in Z-DNA binding were associated with a type I interferon signature [26-28]. Taken together, the data confirmed the biological relevance of the Z-conformation in biology and provided evidence for its role in the regulation of type I interferon responses, both in Mendelian diseases and cancers Questions then arose as to what substrates p150 was targeting, what drove Z-formation in vivo and what pathways the p150 Zα domain regulated.

**The Article by Kim et al**:

The paper by Kim et al. directly addresses the different roles for p150 and p110 in biology, not an easy task given the complexity of dsRNA dependent pathways (Fig. 1B). In a technical tour de force, Kim et al. succeeded in creating a mouse that expressed only p150, but no detectable p110. They did this by knocking out the p110 constitutive promoters and the associated exons 1b and 1c, while leaving the p150 exon 1a and the p150 interferon promoter intact. Previous attempts by these authors and other workers to remove p110 by mutating the initiation codon had failed due to the presence of a downstream methionine that produced a truncated p110. With this new model, the authors were able to directly measure the mRNAs targeted by p150. The other approach to answering the question using p150 Zα mutants were confounded by the expression of p110 [26-28].

The authors found that the p150 only allele was alone sufficient to suppress the interferon signature in mice, even when expressed on a *Adarb1* null background. However, there was an increase in postnatal mortality. The early death phenotype could be reversed by creating a heterozygote with normal p150 expression but with an editing deficient p110 and a p110 null allele, showing editing by p110 is not required for survival. The result confirms that each ADAR isoform has a different role, with p110 playing no role in the interferon response.

The authors examined edited RNAs in the p150 mouse. Overall, RNA editing is dramatically decreased, especially in the brain, occurring at 2% of wildtype levels. Most edits are in the 3' UTR with very few in introns. Recoding of the known substrate AZIN1 is observed, most likely due to formation of a dsRNA substrate within the exon sequence [29]. Together, these findings suggest that p150 editing is mostly cytoplasmic. This makes sense as that is where activation of MDA5 by dsRNAs normally occurs. In contrast, the models with loss of function Zα variants report an overall reduction of Alu-dependent intronic editing, suggesting that p150 can also act in the nucleus. One way to reconcile these findings is that p110 modulates the cellular distribution of p150 between nucleus and cytoplasm, acting as a sponge for cytoplasmic dsRNA that otherwise would block the nuclear uptake of p150. In the absence of p110, p150 stays mostly cytoplasmic. This idea is supported by the finding that nuclear and cytoplasmic levels of p150 and p110 in the Zα loss of function mice are unchanged from wildtype [26].

Figure 1. **A.** The ADAR p150 isoform has the Z-DNA and Z-RNA binding Zα domain that is absent in p110. While p110 is mostly nuclear, p150 undergoes cytoplasmic shuffling. Both isoforms have a bipartite nuclear localization signal (nls) that spans the third double-stranded RNA binding domain 3 (dsRBD3) **B.** The nls is inhibited by double-stranded RNA that can be generated by nuclear export or from cytoplasmic nuclease processing of misfolded or non-coding RNAs. Cytoplasmic dsRNA can also affect other pathways by targeting MDA5 (melanoma differentiation-associated gene 5, activates interferon responses), Stau (Staufen decay pathway) [30], Pkr (protein kinase R, effects on RNA translation) [31] and Oas (2'-5'-oligoadenylate synthetase that activates RNase-L (RNase-L) to cleave non-coding RNAs like tRNA, Y-RNA and ribosomal RNA) [32] **C**. MDA5 forms a filament on dsRNA as shown by the blue blocks bound to the dsRNA helix. It cycles between different states. Hydrolysis of ATP causes dissociation of the filament. In the ATP bound state, MDA5 adopts a more compact conformation as it twists the intervening dsRNA segments as shown by the red arrows. The resulting strain can induce Z-formation [33], most likely by Alu elements [34]. The flip to Z-RNA allows engagement of the ADAR1 p150 Zα domain and the release of MDA5. The process is powered by ATP hydrolysis, converting chemical and mechanical energy to the higher potential energy of Z-RNA. The subsequent edits by p150 then prevent activation of interferon responses by destabilizing the dsRNA. The torsion necessary to induce the Z-RNA flip varies with the length of the dsRNA and the propensity of the embedded sequences to form Z-RNA. Nonself RNAs that lack a Z-forming segment do not engage p150 and instead activate dsRNA dependent responses that protect the host. Those dsRNA viruses that do form Z-RNA undergo genome editing and can also induce necroptosis by activating Z-DNA binding protein1 (ZBP1) [35].

**The future**:

The findings suggest one role for Z-RNA is to protect against interferon induction by self-RNAs (Fig. 1C). Previously it was proposed that MDA5 proof reads dsRNA by using mechanical stress to identify its ligands [36]. In this model, ATP increases the MDA5 filament twist, generating torsion to unwind dsRNA (Fig. 2). Formation of single-stranded RNA triggers MDA5 release and ATP hydrolysis. A different way to relieve the MDA5 induced strain is by Z-RNA formation. The flip to Z lengthens and relaxes the adjacent dsRNA segment by changing its helical pitch from 24.6 Å (A-form) to 45.6 Å (Z-form), triggering MDA5 dissociation. This later cycle uses chemical and mechanical energy to power the conversion of A-form dsRNA to its higher energy Z-RNA conformer.

The sequence class most likely to form dsRNA in the human genome consists of Alu inverted repeats (AIR) that form dsRNA by folding back on themselves. They are able to activate MDA5 dependent interferon responses against self [37]. This outcome is prevented by using p150 editing to destabilize the A-form helix. The Alu repeat family contain highly conserved sequences, referred to as Z-boxes, that are prone to Z-formation [34]. The Z-flip allows AIRs to engage the Zα domain of p150, localizing the deaminase to the adjacent A-form dsRNA. The propensity of Alu dsRNA to form Z-RNA varies with their base composition [34], with the number of AIR in a transcript depending on its length and its processing. These variations impact the editing of 3̊' UTRs, altering the stability and translation of a large number of mRNAs [30, 38]. In addition to Alu, self-RNAs are marked by base modifications. For example, N^6^-methyl adenosine marks restrict MDA5 filament formation [39], potentially leaving some dsRNA segments free to form Z-RNA while changing their propensity to do so [40]. Viruses and other invaders also exploit Z-formation by producing Z-binding proteins [41] that modulate both ADAR p150 dependent editing of their genomes [38] and ZBP1 dependent necroptosis of host cells [35]. Through the work of many, we now have a new understanding of how Z-form nucleic acids impact innate immune responses. Whether Z-RNA formation is induced by MDA5 requires further experimental investigation as does the role of p110 in regulation the p150 cytoplasmic localization. Other helicases, such as DHX9 that targets Alu sequences and associates with p150 [42], may act analogously to MDA5, but power Z-DNA formation. The new findings encourage further exploration of how Z-DNA, Z-RNA and other flipons impact phenotype by changing how genetic information is compiled into programs[40].

Figure 2: The dsRNA helix is shortened in ATP bound MDA5 filaments. The upper figures show MDA5 bound to dsRNA and to either ATP or to the ATP transition state analog ADP-AIF_4_. A cross section of the surface representation of the protein allows visualization of the bound dsRNA and the nucleotide ligand (on the right of each image). The lower figures show only one strand of the RNA double-helix (dsRNA) from the cryo-EM structures described in PDB records 6GKM and 6GKH to emphasize the difference in the relative position of the RNA base 14 in the two complexes due to differences how the filament twists [36]. The compact MDA5 complex in the ATP bound has 5 MDA5 subunits per step, corresponding to a twist of ~ 72 ̊ for each. The ADP-AIF_4_ complex has 4 subunits per step, corresponding to a twist ~ 90̊ for each [36]. The extra twist of the ATP filament creates torsion of the adjacent dsRNA that can be relieved by unwinding the dsRNA into single-stranded RNAs [36]. Under the model described here, the strain is released by flipping the right-handed A-form dsRNA to the longer left-handed Z-RNA conformation, triggering ATP hydrolysis as MDA5 releases.

**References**

1.	Wang AH, Quigley GJ, Kolpak FJ, Crawford JL, van Boom JH, van der Marel G, et al. Molecular structure of a left-handed double helical DNA fragment at atomic resolution. Nature. 1979;282(5740):680-6. Epub 1979/12/13. PubMed PMID: 514347.

2.	Pohl FM, Jovin TM. Salt-induced co-operative conformational change of a synthetic DNA: equilibrium and kinetic studies with poly (dG-dC). J Mol Biol. 1972;67(3):375-96. Epub 1972/06/28. PubMed PMID: 5045303.

3.	Peck LJ, Wang JC. Energetics of B-to-Z transition in DNA. Proc Natl Acad Sci U S A. 1983;80(20):6206-10. Epub 1983/10/01. PubMed PMID: 6578505; PubMed Central PMCID: PMC394264.

4.	Herbert A, Lowenhaupt K, Spitzner J, Rich A. Double-stranded RNA adenosine deaminase binds Z-DNA in vitro. Nucleic Acids Symp Ser. 1995;(33):16-9. Epub 1995/01/01. PubMed PMID: 8643357.

5.	Bass BL. RNA editing by adenosine deaminases that act on RNA. Annu Rev Biochem. 2002;71:817-46. Epub 2002/06/05. doi: 10.1146/annurev.biochem.71.110601.135501. PubMed PMID: 12045112; PubMed Central PMCID: PMC1823043.

6.	Sommer B, Köhler M, Sprengel R, Seeburg PH. RNA editing in brain controls a determinant of ion flow in glutamate-gated channels. Cell. 1991;67(1):11-9. doi: 10.1016/0092-8674(91)90568-j.

7.	Higuchi M, Maas S, Single FN, Hartner J, Rozov A, Burnashev N, et al. Point mutation in an AMPA receptor gene rescues lethality in mice deficient in the RNA-editing enzyme ADAR2. Nature. 2000;406(6791):78-81. Epub 2000/07/14. doi: 10.1038/35017558. PubMed PMID: 10894545.

8.	Patterson JB, Thomis DC, Hans SL, Samuel CE. Mechanism of interferon action: double-stranded RNA-specific adenosine deaminase from human cells is inducible by alpha and gamma interferons. Virology. 1995;210(2):508-11. Epub 1995/07/10. doi: 10.1006/viro.1995.1370. PubMed PMID: 7618288.

9.	Barraud P, Banerjee S, Mohamed WI, Jantsch MF, Allain FH. A bimodular nuclear localization signal assembled via an extended double-stranded RNA-binding domain acts as an RNA-sensing signal for transportin 1. Proc Natl Acad Sci U S A. 2014;111(18):E1852-61. Epub 2014/04/23. doi: 10.1073/pnas.1323698111. PubMed PMID: 24753571; PubMed Central PMCID: PMCPMC4020056.

10.	Sakurai M, Shiromoto Y, Ota H, Song C, Kossenkov AV, Wickramasinghe J, et al. ADAR1 controls apoptosis of stressed cells by inhibiting Staufen1-mediated mRNA decay. Nat Struct Mol Biol. 2017;24(6):534-43. Epub 2017/04/25. doi: 10.1038/nsmb.3403. PubMed PMID: 28436945; PubMed Central PMCID: PMCPMC5461201.

11.	Athanasiadis A, Rich A, Maas S. Widespread A-to-I RNA editing of Alu-containing mRNAs in the human transcriptome. PLoS Biol. 2004;2(12):e391. Epub 2004/11/10. doi: 10.1371/journal.pbio.0020391. PubMed PMID: 15534692; PubMed Central PMCID: PMC526178.

12.	Kim DD, Kim TT, Walsh T, Kobayashi Y, Matise TC, Buyske S, et al. Widespread RNA editing of embedded alu elements in the human transcriptome. Genome Res. 2004;14(9):1719-25. Epub 2004/09/03. doi: 10.1101/gr.2855504. PubMed PMID: 15342557; PubMed Central PMCID: PMC515317.

13.	Schaffer AA, Levanon EY. ALU A-to-I RNA Editing: Millions of Sites and Many Open Questions. Methods Mol Biol. 2021;2181:149-62. Epub 2020/07/31. doi: 10.1007/978-1-0716-0787-9_9. PubMed PMID: 32729079.

14.	Wang Q, Khillan J, Gadue P, Nishikura K. Requirement of the RNA editing deaminase ADAR1 gene for embryonic erythropoiesis. Science. 2000;290(5497):1765-8. Epub 2000/12/02. PubMed PMID: 11099415.

15.	Liddicoat BJ, Piskol R, Chalk AM, Ramaswami G, Higuchi M, Hartner JC, et al. RNA editing by ADAR1 prevents MDA5 sensing of endogenous dsRNA as nonself. Science. 2015;349(6252):1115-20. Epub 2015/08/15. doi: 10.1126/science.aac7049. PubMed PMID: 26275108; PubMed Central PMCID: PMC5444807.

16.	Mannion NM, Greenwood SM, Young R, Cox S, Brindle J, Read D, et al. The RNA-editing enzyme ADAR1 controls innate immune responses to RNA. Cell Rep. 2014;9(4):1482-94. Epub 2014/12/03. doi: 10.1016/j.celrep.2014.10.041. PubMed PMID: 25456137; PubMed Central PMCID: PMC4542304.

17.	Ward SV, George CX, Welch MJ, Liou LY, Hahm B, Lewicki H, et al. RNA editing enzyme adenosine deaminase is a restriction factor for controlling measles virus replication that also is required for embryogenesis. Proc Natl Acad Sci U S A. 2011;108(1):331-6. Epub 2010/12/22. doi: 10.1073/pnas.1017241108. PubMed PMID: 21173229; PubMed Central PMCID: PMC3017198.

18.	Pestal K, Funk CC, Snyder JM, Price ND, Treuting PM, Stetson DB. Isoforms of RNA-Editing Enzyme ADAR1 Independently Control Nucleic Acid Sensor MDA5-Driven Autoimmunity and Multi-organ Development. Immunity. 2015;43(5):933-44. Epub 2015/11/21. doi: 10.1016/j.immuni.2015.11.001. PubMed PMID: 26588779; PubMed Central PMCID: PMC4654992.

19.	Rice GI, Kasher PR, Forte GM, Mannion NM, Greenwood SM, Szynkiewicz M, et al. Mutations in ADAR1 cause Aicardi-Goutieres syndrome associated with a type I interferon signature. Nat Genet. 2012;44(11):1243-8. Epub 2012/09/25. doi: 10.1038/ng.2414. PubMed PMID: 23001123; PubMed Central PMCID: PMC4154508.

20.	Herbert A. Mendelian Disease caused by variants affecting recognition of Z-DNA and Z-RNA by the Zα domain of the double-stranded RNA Editing Enzyme ADAR. European Journal of Human Genetics. 2019. doi: 10.1038/s41431-019-0458-6.

21.	Schade M, Turner CJ, Lowenhaupt K, Rich A, Herbert A. Structure-function analysis of the Z-DNA-binding domain Zalpha of dsRNA adenosine deaminase type I reveals similarity to the (alpha + beta) family of helix-turn-helix proteins. Embo J. 1999;18(2):470-9. Epub 1999/01/16. doi: 10.1093/emboj/18.2.470. PubMed PMID: 9889202; PubMed Central PMCID: PMC1171140.

22.	Schade M, Turner CJ, Kuhne R, Schmieder P, Lowenhaupt K, Herbert A, et al. The solution structure of the Zalpha domain of the human RNA editing enzyme ADAR1 reveals a prepositioned binding surface for Z-DNA. Proc Natl Acad Sci U S A. 1999;96(22):12465-70. Epub 1999/10/27. PubMed PMID: 10535945; PubMed Central PMCID: PMC22950.

23.	Schwartz T, Rould MA, Lowenhaupt K, Herbert A, Rich A. Crystal structure of the Zalpha domain of the human editing enzyme ADAR1 bound to left-handed Z-DNA. Science. 1999;284(5421):1841-5. Epub 1999/06/12. PubMed PMID: 10364558.

24.	Placido D, Brown BA, 2nd, Lowenhaupt K, Rich A, Athanasiadis A. A left-handed RNA double helix bound by the Z alpha domain of the RNA-editing enzyme ADAR1. Structure. 2007;15(4):395-404. Epub 2007/04/18. doi: 10.1016/j.str.2007.03.001. PubMed PMID: 17437712; PubMed Central PMCID: PMC2082211.

25.	Herbert A. ADAR and Immune Silencing in Cancer. Trends Cancer. 2019;5(5):272-82. Epub 2019/06/09. doi: 10.1016/j.trecan.2019.03.004. PubMed PMID: 31174840.

26.	de Reuver R, Dierick E, Wiernicki B, Staes K, Seys L, De Meester E, et al. ADAR1 interaction with Z-RNA promotes editing of endogenous double-stranded RNA and prevents MDA5-dependent immune activation. 2020. doi: 10.1101/2020.12.04.411702.

27.	Tang Q, Rigby RE, Young GR, Korning-Hvidt A, Tan TK, Bridgeman A, et al. Recognition of Z-RNA by ADAR1 limits interferon responses. 2020. doi: 10.1101/2020.12.04.411793.

28.	Nakahama T, Kato Y, Shibuya T, Kim JI, Vongpipatana T, Todo H, et al. Disruption of Z-RNA–binding of ADAR1 induces Aicardi-Goutières syndrome–like encephalopathy in mice. 2020. doi: 10.1101/2020.12.16.422984.

29.	Costa Cruz PH, Kato Y, Nakahama T, Shibuya T, Kawahara Y. A comparative analysis of ADAR mutant mice reveals site-specific regulation of RNA editing. Rna. 2020;26(4):454-69. Epub 2020/01/17. doi: 10.1261/rna.072728.119. PubMed PMID: 31941663; PubMed Central PMCID: PMCPMC7075269.

30.	Maquat LE. Short interspersed nuclear element (SINE)-mediated post-transcriptional effects on human and mouse gene expression: SINE-UP for active duty. Philos Trans R Soc Lond B Biol Sci. 2020;375(1795):20190344. Epub 2020/02/23. doi: 10.1098/rstb.2019.0344. PubMed PMID: 32075563; PubMed Central PMCID: PMCPMC7061979.

31.	Samuel CE. Adenosine deaminase acting on RNA (ADAR1), a suppressor of double-stranded RNA-triggered innate immune responses. J Biol Chem. 2019;294(5):1710-20. Epub 2019/02/03. doi: 10.1074/jbc.TM118.004166. PubMed PMID: 30710018.

32.	Donovan J, Rath S, Kolet-Mandrikov D, Korennykh A. Rapid RNase L–driven arrest of protein synthesis in the dsRNA response without degradation of translation machinery. Rna. 2017;23(11):1660-71. doi: 10.1261/rna.062000.117.

33.	Ivanov IE, Lebel P, Oberstrass FC, Starr CH, Parente AC, Ierokomos A, et al. Multimodal Measurements of Single-Molecule Dynamics Using FluoRBT. Biophys J. 2018;114(2):278-82. Epub 2017/12/19. doi: 10.1016/j.bpj.2017.11.017. PubMed PMID: 29248150; PubMed Central PMCID: PMCPMC5984952.

34.	Herbert A. Z-DNA and Z-RNA in human disease. Communications Biology. 2019;2(1):7. doi: 10.1038/s42003-018-0237-x.

35.	Zhang T, Yin C, Boyd DF, Quarato G, Ingram JP, Shubina M, et al. Influenza Virus Z-RNAs Induce ZBP1-Mediated Necroptosis. Cell. 2020;180(6):1115-29 e13. Epub 2020/03/24. doi: 10.1016/j.cell.2020.02.050. PubMed PMID: 32200799; PubMed Central PMCID: PMCPMC7153753.

36.	Yu Q, Qu K, Modis Y. Cryo-EM Structures of MDA5-dsRNA Filaments at Different Stages of ATP Hydrolysis. Mol Cell. 2018;72(6):999-1012 e6. Epub 2018/11/20. doi: 10.1016/j.molcel.2018.10.012. PubMed PMID: 30449722; PubMed Central PMCID: PMCPMC6310684.

37.	Ahmad S, Mu X, Yang F, Greenwald E, Park JW, Jacob E, et al. Breaching Self-Tolerance to Alu Duplex RNA Underlies MDA5-Mediated Inflammation. Cell. 2018;172(4):797-810 e13. Epub 2018/02/06. doi: 10.1016/j.cell.2017.12.016. PubMed PMID: 29395326; PubMed Central PMCID: PMC5807104.

38.	Pfaller CK, Donohue RC, Nersisyan S, Brodsky L, Cattaneo R. Extensive editing of cellular and viral double-stranded RNA structures accounts for innate immunity suppression and the proviral activity of ADAR1p150. PLoS Biol. 2018;16(11):e2006577. Epub 2018/11/30. doi: 10.1371/journal.pbio.2006577. PubMed PMID: 30496178; PubMed Central PMCID: PMC6264153.

39.	Shulman Z, Stern-Ginossar N. The RNA modification N(6)-methyladenosine as a novel regulator of the immune system. Nat Immunol. 2020;21(5):501-12. Epub 2020/04/15. doi: 10.1038/s41590-020-0650-4. PubMed PMID: 32284591.

40.	Herbert A. A Genetic Instruction Code Based on DNA Conformation. Trends Genet. 2019. Epub 2019/11/02. doi: 10.1016/j.tig.2019.09.007. PubMed PMID: 31668857.

41.	Athanasiadis A. Zalpha-domains: At the intersection between RNA editing and innate immunity. Seminars in Cell & Developmental Biology. 2012;23(3):275-80. doi: 10.1016/j.semcdb.2011.11.001.

42.	Aktas T, Avsar Ilik I, Maticzka D, Bhardwaj V, Pessoa Rodrigues C, Mittler G, et al. DHX9 suppresses RNA processing defects originating from the Alu invasion of the human genome. Nature. 2017;544(7648):115-9. Epub 2017/03/30. doi: 10.1038/nature21715. PubMed PMID: 28355180.
